# Lysosome purinergic receptor P2X4 regulates neoangiogenesis induced by microvesicles from sarcoma patients

**DOI:** 10.1038/s41419-021-04069-w

**Published:** 2021-08-17

**Authors:** Wulf Palinski, Maria Monti, Rosa Camerlingo, Ilaria Iacobucci, Serena Bocella, Federica Pinto, Clara Iannuzzi, Gelsomina Mansueto, Sara Pignatiello, Flavio Fazioli, Michele Gallo, Laura Marra, Flora Cozzolino, Annarosaria De Chiara, Piero Pucci, Antonio Bilancio, Filomena de Nigris

**Affiliations:** 1 Department of Medicine, University of California San Diego, La Jolla, CA USA; 2 Department of Chemical Sciences, University of Napoli Federico II and CEINGE Advanced Biotechnologies, Naples, Italy; 3 Department of Cell Biology and Biotherapy Research, Istituto Nazionale Tumori IRCCS - Fondazione G. Pascale, Naples, Italy; 4 Department of Experimental Medicine, University of Campania “LuigiVanvitelli”, Naples, Italy; 5 Department of Precision Medicine, University of Campania “LuigiVanvitelli”, Naples, Italy; 6 Department of Advanced Biomedical Sciences, University of Naples Federico II, Naples, Italy; 7 Department of Advanced Medical and Surgical Sciences, University of Campania “Luigi Vanvitelli”, Naples, Italy; 8 Division of Skeletal Muscle Oncology Surgery, Istituto Nazionale Tumori IRCCS - Fondazione G. Pascale, Naples, Italy; 9 Division of Anatomy, Istituto Nazionale Tumori IRCCS - Fondazione G. Pascale, Naples, Italy

**Keywords:** Cancer microenvironment, Cell polarity

## Abstract

The tumor microenvironment modulates cancer growth. Extracellular vesicles (EVs) have been identified as key mediators of intercellular communication, but their role in tumor growth is largely unexplored. Here, we demonstrate that EVs from sarcoma patients promote neoangiogenesis via a purinergic X receptor 4 (P2XR4) -dependent mechanism in vitro and in vivo. Using a proteomic approach, we analyzed the protein content of plasma EVs and identified critical activated pathways in human umbilical vein endothelial cells (HUVECs) and human progenitor hematopoietic cells (CD34+). We then showed that vessel formation was due to rapid mitochondrial activation, intracellular Ca^2+^ mobilization, increased extracellular ATP, and trafficking of the lysosomal P2XR4 to the cell membrane, which is required for cell motility and formation of stable branching vascular networks. Cell membrane translocation of P2XR4 was induced by proteins and chemokines contained in EVs (e.g. Del-1 and SDF-1). Del-1 was found expressed in many EVs from sarcoma tumors and several tumor types. P2XR4 blockade reduced EVs-induced vessels in angioreactors, as well as intratumor vascularization in mouse xenografts. Together, these findings identify P2XR4 as a key mediator of EVs-induced tumor angiogenesis via a signaling mediated by mitochondria-lysosome-sensing response in endothelial cells, and indicate a novel target for therapeutic interventions.

## Introduction

Cancer cells release more extracellular vesicles (EVs) than normal cells, leading to higher concentrations in patient blood [1, 2]. Several studies suggest that they are absorbed on the surface of target cells and penetrate them. Tumor EVs play multiple roles in paracrine and autocrine cell communication and in the regulation of molecular pathways of malignancies [3–7]. EVs derived from activated platelets contribute to metastasis of lung cancer [5]. EVs from renal carcinoma stem cells prepare metastatic niche [8] and transfer Pyruvate kinase M2 (PKM2) into different cell types of the microenvironment, favoring hepatocarcinoma progression [3]. Based on multiple studies reporting molecular pathways activated by EVs, they are also considered a “source of biomarkers” [9]. However, given the histological and genetic heterogeneity of malignancies, the composition of tumor EVs varies and their role remains largely unexplored [10].

Neoangiogenesis plays an important role in cancer growth. Tumor EVs contain a variety of proangiogenic factors [11, 12], which are a prominent target of cancer therapy. Bone cancers comprise several highly vascularized, drug-resistant subtypes [13–17]. We therefore investigated angiogenic mechanisms of cancers triggered by large EVs from giant cell tumors of bone (GCTB), which represent ~5% of all primary bone tumors and constitute a well-defined clinicopathological and molecular entity [11, 12]. Histologically, GCTB are composed of neoplastic mononuclear cells of myeloid linage and multinucleated osteoclast-like giant cells [13, 14]. Treatment is based essentially on Denosumab antibody against RANKL [18] and novel immune-based regimens are emerging from three-dimensional models of individual biopsies [19, 20].

Here we assess the role of large EVs, from blood of sarcoma patients, on the formation of new vessels and identify a lysosomal receptor P2XR4-dependent signaling mechanism promoting angiogenesis by influencing endothelial mitochondrial activity and cell motility.

## Material and methods

### Cell cultures and microvesicles (MVs) preparation

Cells from biopsies were obtained by digestion with collagenase type IV (0.2 mg/ml, Gibco, Italy) for 1 h at 37 °C and grown in RPMI 1640 medium with 10% FBS, l-glutamine, and penicillin/streptomycin (Life Technologies, Milan, Italy). Human umbilical vein endothelial cells (HUVECs) from Lonza (Milan Italy) were grown in basal medium (EGM2, Lonza) enriched with SingleQuots^TM^. All experiments were performed under cell passages 2–6. CD34+ cells were isolated from 20 ml of healthy donors’ blood apheresis product, stratified on Ficoll-Paque PLUS (Histopaque 1077GE Healthcare Bio-Sciences) and enriched by two runs of immunomagnetic selection on CD34+ and CD133+ MiniMACS columns (Miltenyi Biotec, Gladbach, Germany) in accordance with the manufacturer’s instructions.

### Isolation and characterization of EVs

EVs from biopsy culture cells were obtained from conditioned media of 10^7^ cells at 80% of confluence, and grown with 10% Exo-FBS for 24 h (FBS depleted of exosomes, SBI, System Bioscience). Plasma EVs from tumor patients were isolated from 10 ml of blood collected during surgery or from 10 ml of blood from healthy donors. Conditioned media and plasmas were once centrifugated for 10 min at 4 °C and 400 × *g*, followed by three times at 5000 × *g*. The supernatant was then ultracentrifugated twice at 14,000 × *g* for 35 min at 4 °C in a Beckman ultracentrifuge with Ti70 rotor [21]. The resulting pellets were resuspended in PBS with 3.2% NaCitrate (0.11 M) and 1× protease inhibitor cocktail (Sigma-Aldrich) and stored at −80 °C. Equal amounts of MV proteins (quantified by Bradford assay) were used in all functional assays.

### Determination of particle number and size distribution

Particle diameters of the EVs fractions in a range between 0 and 1000 nm were analyzed in 3D by Zeta sizer nano 25 ZSP (Malvern Panalytical, Malvern, UK). The size and concentration of EVs were determined by NanoSight NS300 instrument (Malvern Instruments, UK). Different sample dilutions (1:50 to 1:2000, in PBS) with particle concentration in the optimal detection range (5 × 10^8^ to 1 × 10^9^ particles/ml) were determined. Camera settings were kept constant during all acquisitions of the same experiment: Camera level, 7–9; camera gain, 10–12; detection threshold, 2–4. Particle numbers and sizes were calculated based on the Stokes–Einstein equation.

### Tube formation assays

HUVECs (6 × 10^4^ cells) were grown in μslide IbiDI culture plates with reduced matrigel and the reaction was stopped after 18 h of stimulation. A total of 1 × 10^4^ CD34+ cells were grown in μslide plates IbiDI with 10 ng/ml fibronectin in EGM2 medium with 0.2% serum, then preincubated for 1 h with 10 μg/ml anti-KDR antibody (R&D Systems, Minneapolis, USA) or 100 ng/ml Bevacizumab (Avastin, Roche), and then stimulated with 6 μg/ml of patient MVs or heat inactivated by 10 min at 80 °C. Images were captured by Zeiss confocal microscope and microvessel lengths and branch numbers quantified by ZEN software.

### Transfection SIRNAs

P2X4R mRNA and protein expression in HUVECs was silenced by siRNATrilencer-27 transfection for 48 h with 5 nM P2X4R siRNA or 5 nM scrambled siRNA (SR303323 OriGene Technologies; Rockville, MD, USA), along with jetPRIME transfection reagent (Polypus transfection™; Bioparc, France). For more details see Supplementary methods.

### Flow cytometry

Fifty micrograms of MVs was diluted 1:50 in PBS and incubated for 30 min at room temperature with specific antibodies, or stained with 2 μg/ml of 7-aminoactinomycin D (7-AAD) (A1310 Thermo Fisher), in PBS buffer containing 3.2% NaCitrate (0.11 M) and 1× protease inhibitor cocktail (Sigma-Aldrich). Microparticles (0.1, 0.5, 1, and 2 μm) were used to calibrate FACS sorting (Polysciences, Warrington, PA). Cells and MVs were sorted by FACS ARIA III (Becton Dickinson, Franklin Lakes, NJ). At least 30,000 events were analyzed at each experimental point. Control experiments were performed with isotype-matched human IgG (Becton Dickinson) (for antibodies used see extended methods).

### Confocal immunofluorescence

A total of 2 × 10^4^ HUVECs or CD34+ cells were grown on glass coverslips (IbiDI), fixed in 4% paraformaldehyde, permeabilized with 0.1% Triton, and incubated for 12 h at 4 °C with mouse monoclonal antibodies to human CD31 (Agilent DAKO), LAMP-1 (MA5-28267 Thermo Fisher), rabbit anti P2XR4 (ALX-215-034 Enzo Life Science) or mouse anti-ICAM-1 antibodies (Agilent DAKO). Cells were then stained by addition of secondary antibodies conjugated with Alexa-488, Alexa-568, or Alexa-647 (1:1000; Molecular Probes, Thermo Fisher). Fluorescence images were captured through 20×, 63×, or 100× oil objectives, using Zeiss microscope imaging software. Confocal images were analyzed by Zeiss image analyzer (version 7.2.3; Bit plane). Intensities of different fluorophores were correlated by Pearson’s coefficient. The colocalization between intensity of different fluorophores was quantified using the Manders’ algorithm.

### Intracellular calcium, mitochondrial redox status, and microscopy

For ATP measurements, 5 × 10^4^ CD34+ cells or HUVECs were plated in 24 multiwell plates preincubated for 5 min with inhibitors CBX (50 μM), Pannexin1 (10 μM), 5-BDBD (5 μM), CCCP (5 μM) and Apyrase (20 U), stimulated for 1 min or 10 min with 6 μg/ml MVs, and Del-1, then assayed with ATP bioluminescence kit (Thermo Fisher Scientific). Mitochondrial activity was assayed by adding 10 nM MitoTracker CM-H2XRos (Thermo-Fisher M7513) to the cell media, and after washing the fluorescence was measured in living cells by TECAN infinity 2000 (Ex 579 /Em599). Mitochondrial morphology was determined with 10 nM MitoTracker Green or Red (Termo-Fisher 754). Ca^2+^ measurement was carried out in HUVECs loaded with 5 mM Fura-2 AM, using TECAN infinity 2000 system. Changes in intracellular Ca^2+^ are represented as the ratio of Fura-2 AM fluorescence induced at an emission wavelength of 510 nm and excitation at 340 and 380 nm (ratio = F340/F380). Experiments were done in free Ca^2+^ solution (in mM: 140 NaCl, 2.7 KCl, 4 MgCl_2_, 0.5 EGTA, 10 HEPES, pH 7.4), and Ca^2+^ influx was determined from changes in Fura-2 fluorescence after re-addition of Ca^2^ (2.5 mM). HUVECs were stimulated 5 min with Ionomicyn (5 nM) and ATP (50 μM) as controls.

### Fluorescence lifetime imaging microscopy (FLIM)

FLIM was used to estimate the fluorescence lifetime of the molecular rotor 4,4-difluoro-5,7-dimethyl-4-bora-3a,4a-diaza-s-indacene-3-dodecanoic acid (Bodipy FL C12) [22]. FLIM was performed with an ISS Alba frequency domain confocal FLIM microscope (ISS, Champaign, IL), at water immersion objective (60×, N.A. = 1.2). Fluorescence lifetime (*τ*) values are expressed as nanoseconds (ns), as mean ± SEM of three independent measurements. Pixel fits to the lifetime data were performed using the manufacturer’s software (Vista Vision 4.0).

### Proteomic analysis

Thirty micrograms of proteins for each condition was digested by trypsin onto S-Trap filters, following the protein digestion protocol of the manufacturer (Protifi, Huntington, NY). Two biological replicates were analyzed for individual MVs, three for CD34+ cells treated for 30 min versus control group, and two for cells treated for 24 h versus control group. Each biological replicate was analyzed in duplicate by nano LC–MS/MS, using the Easy-nLC II chromatographic system coupled with a linear trap quadropole (LTQ) Orbitrap XL mass spectrometer (Thermo Fisher Scientific, Waltham, MA). The fold changes were calculated as label free quantification (LFQ) values, using Viewer 26 to reveal significantly changed proteins. Proteins were identified by three peptides. Proteins were then further analyzed by Clue-Go plug-in, in the latest version of Cytoscape software (3.7.1). Protein accession numbers and Gene Ontology (GO) database were used to cluster data according to cellular components and pathways (see Supplementary methods).

### RT-PCR

Five hundred nanograms of total RNA was converted into cDNA, using a Transcriptor First Strand cDNA synthesis kit (Roche, Penzberg, Germany). For primers used for real-time and condition see Supplementary methods. Data were determined by the 2^−Δ/ΔCt^ method.

### Western blots

Thirty micrograms of protein extracts was resolved by SDS-PAGE, transferred to nitrocellulose membranes, and incubated overnight at 4 °C with CD9- or HSP70-specific antibodies in 1% BSA (Ts9 Cat #10626D Invitrogen, 3A3 Cat #MA3-006 Thermo-Fisher) and Tubulin monoclonal antibody (Cat# OAPA00361 Aviva System). Antigen-bound antibodies were visualized by ECL. Uncropped immunoblots areas of the main figures are shown in supplements, as are other antibod used.

### Direct in vivo angiogenesis assay (DIVA) and xenograph

#### Tumor growth

A total of 1 × 10^6^ Saos cells in 100 μl were injected into the flank of 6-week-old female athymic (nude/nude) CD-1 mice (ENVIGO Laboratories) (randomly distributed in four groups and *n* = 5 per group). One group receiving only T-MVs, the second T-MVs +5-BDBD, the third group C-MVs, the four group C-MVs+ 5-BDBD. Starting on day 7 after cell implantation until day 21, mice were injected intraperitoneally 3 times per week with 5-BDBD at a dose of 4.25 mg/kg, or 70 μg T-MVs-PHK23 or 70 μg C-MVs-PHK23, in a volume of 100 μl. Tumor diameters were measured using a caliper, and the volumes were calculated using the formula: Volume =  Length × (Width)^2^, where the ‘length’ corresponds to the longest and the ‘width’ to the shortest of the measured tumor diameters.

#### DIVA

In vivo angiogenesis was also determined by DIVA (3450- 048-K, R&D Systems). Angioreactors were filled with basement membrane extract (10 μl) premixed with 6 μg T-MVs, C-MVs, or 1 μM Del-1 with or without 5-BDBD while for genetic silencing of P2XR4, 5 nM of scramble siRNA or siP2XR4 were added to T-MVs treatment (see Supplementary methods). Four Angioreactors were implanted in different mouse groups (*n* = 3 per group) and injected intraperitoneally three times per week from day 1 to day 14 post implantation with 5-BDBD at a dose of 4.25 mg/kg, or 70 μg T-MVs-PHK23 or 70 μg C-MVs-PHK23, 1 nmol (around 0.4 mg/kg) of scramble siRNA or siP2XR4-RNA in a volume of 100 μl; at day 14 the angioreactors were explanted and quantified by FITC lectin at 550 nm.

### Retinal preparation

P6 old mice (*n* = 3 per group) were intraperitoneus injected with 100 μl of 70 μg/ml of MVs or 100 ng/ml of VEGF. At P10 mice retinae were prepared by dissecting eyes, fixing them in 4% paraformaldehyde for 15 min at RT. After removing the cornea, sclera, lens, and hyaloids vessels, retinas were fixed in methanol at −20 °C for 12 h, and permeabilized in blocking buffer (1% BSA) and 3% Triton X-100 in PBS for 3 h at 4 °C. For immunostaining, I-isolectin B4 (IB4, P5704 Sigma) was diluted in 0.3% Triton X-100, 1 mMCaCl_2_, 1 mM MnCl_2_ and 1 mM MgCl_2_ in PBS, pH 6.8, and retinas were stained overnight at 4 °C. Retinas were flat-mounted with Moviol and images obtained with Zeiss microsope and imageJ software (National Institutes of Health, MD, USA).

### Animal use

All animal experiments were performed in compliance with the Italian GPL Guidelines (Italian Law Decree 116/92 issued by the Ministry of Health) and Directive 201/63/EU of the European Parliament on the protection of animals used for scientific purposes. Protocols relating to the present work were approved by the Animal Care and Use Committee of the University of Campania “L. Vanvitelli”, Naples, Italy. Animals were acclimatized, quarantined for at least 1 week and then housed in micro-controlled individual cages with ad libitum access to food and water. All efforts were made to minimize animal suffering and to reduce the number of animals.

### Statistical analysis

Statistical analysis was performed by Student’s t-test, one-way ANOVA, non-parametric Mann–Whitney *T*-test, post-hoc Tukey’s tests, or Heatmap using SPSS version 21. Sample sizes were chosen based on previous experience. Differences were considered significant at P < 0.05.

Special materials and reagents are listed in Supplementary data.

## Results

### Characteristics of tumor extracellular microvesicles derived from plasma of sarcoma patients

**Fig. 1:**
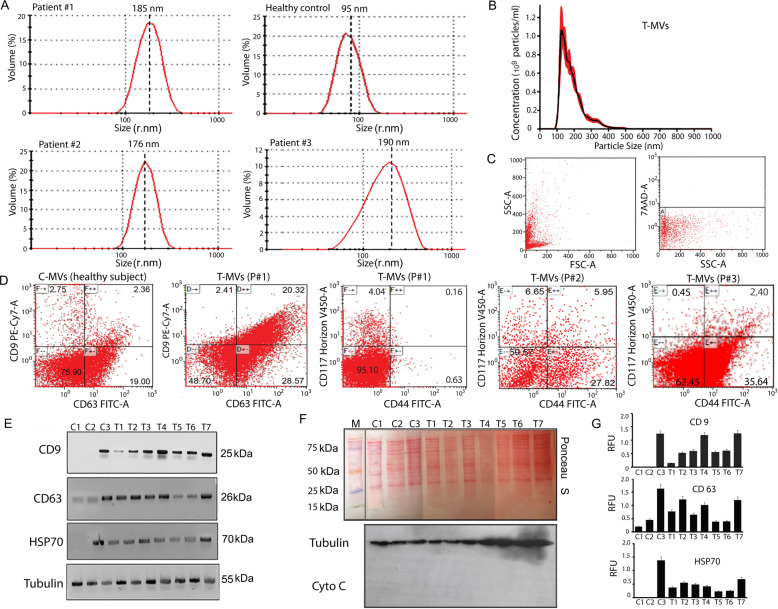
Characterization of tumor microvesicles. **A** Particle volume distribution in function of sizes of plasma tumor MVs (T-MVs) from giant cell tumor of bone patient 1, healthy control microvesicles (C-MVs), T-MVs from patient 2, and patient 3, as indicated. **B** Distribution of particle concentrations in function of sizes in a representative preparation of plasma T-MVs determined by nanoparticle-tracking analysis (NanoSight). Histogram of typical preparations containing 10^9^ T-MVs/ml. **C** Flow cytometry scatter plot of T-MVs showing autofluorescence settings and staining with 7-AAD, indicating high integrity of isolated MVs. **D** Representative fluorescence activated cell sorting (FACS) analysis of healthy subjects’ control MVs (C-MVs) and T-MVs detected by CD63 and CD9 antibodies and cytometric analysis of CD44, and CD117 antigen expression in T-MVs of different patients. Percent of positive MVs are indicated in each quadrant. **E** Western blots of 30 µg protein extracts from isolated MVs. Lanes C1–C3; proteins from healthy control microvesicles (C-MVs). Lanes T1–T7, proteins from tumor patient microvesicles (T-MVs). Specific antibodies used for immunoblots are indicated. **F** Upper panel: red PONCEAU blots of 30 µg protein extracts from isolated MVs. Lane M; marker, Lanes C1–C3; proteins from healthy control microvesicles (C-MVs). Lanes T1–T7, proteins from tumor patient microvesicles (T-MVs). Lower panel western blot for Cytochrome C determination (negative control). Tubulin was used as load control. **G** Quantification of western blots shown in (**E**). Relative expression was determined using Image J software.

Large tumor microvesicles (T-MVs) were prepared from the plasma of six different GCTB patients, three healthy donors and one from cultured media of tumor biopsy using differential ultracentrifugation [21]. Representative preparation of T-MVs had a diameter distribution range of 100–250 nm radiant (measured also considering angular dimensions *X*, *Y*, *Z*) with a major peak at ~150 nm (Fig. 1A and Supplementary Table S1), confirmed by nanoparticle-tracking analysis (Fig. 1B). Control microvesicles (C-MVs) from healthy subjects prepared in the same condition had smaller diameters (Fig. 1A). Clinical pathological characteristics of patients and their MVs sizes are shown in Table S1. Specific MVs markers for studies of EVs required by the guideline of the International Society for Extracellular Vesicles (MISEV2018) were detected [23]. Fluorescence cell sorting analysis showed that plasma T-MVs were positive for CD9 and CD63 antigens (category-1 EV markers) ranging between 1 and 20 percent. A representative analysis is shown in Fig. 1C, D. T-MVs also expressed antigens typical of mesenchymal cells, i.e., CD90, CD117, and CD44, in a range varying from patient to patient (Fig. 1D). By western blots we also detected CD9, CD63, HSP70 (a category-2 stress marker), and tubulin (a category-2b EV marker) proteins (Fig. 1E) in both control MVs and T-MVs. All large MVs prepared were negative to cytochrome C, considered apoptotic body markers, although we cannot exclude that exosomes were also present in MVs preparation.

### Tumor-derived microvesicles are proangiogenic

**Fig. 2:**
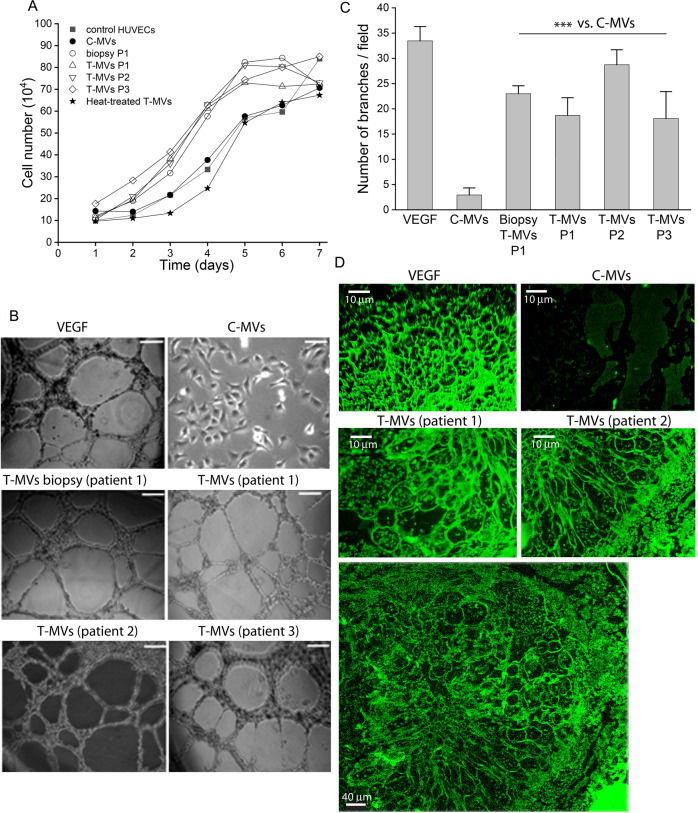
Tumor MVs promote branch formation in HUVECs and murine retinas. **A** Proliferation of HUVECs cultured for 7 days in medium enriched with MVs of healthy subject (C-MVs) or tumor patients (T-MVs) isolated from plasma or media of biopsy cell cultures (biopsy P1) and heat inactivated T-MVs (as indicated). **B** Representative images of branches formed by 10^4^ HUVECs in matrigel μSlide ibidi stimulated for 18 h with 6 μg/ml of T-MVs from different patients, C-MVs from a healthy patient or 10 ng/ml VEGF. Scale bars = 100 μm. **C** Number of branches per visual field at ×10 of magnification formed in HUVECs treated with 10 ng/ml VEGF or 6 μg/ml of control microvesicles (C-MVs), or T-MVs prepared from media of cultured biopsy cells and tumor microvesicles (T-MVs) from patients P1, P2, and P3. The experiment was performed in triplicate. Data are mean ± SD. ***P < 0.001. v.s. C-MVs. **D** Representative images of whole-mount retinas from P10 mice (*n* = 3 per group) treated with 100 ng/ml VEGF, 6 μg/ml of C-MVs, or T-MVs of two patients, and stained with FITC isolectin B4, (Scale bars = 10 μm). Lower panel: higher magnification showing dilated vessels in the vascular plexus and numerous glomerular capillaries throughout the retina of a mouse treated with T-MVs of patient 2. (Scale bar = 40 μm).

To test the role of T-MVs in neoangiogenesis, a branch-formation assay was performed testing different MVs (protein) concentrations (Supplementary Fig. 1). In HUVECs, T-MVs from some patients (3 GCTB patients) promoted branching networks, which was abolished by heat inactivation. No such effect was observed with C-MVs from healthy individuals at any dosage tested (Fig. 2A and Supplementary Fig. 1). Because myeloid cells are components of giant cell tumor histology, we also used T-MVs to stimulate human hematopoietic progenitor cells (CD34+) isolated from healthy donors (see “Material and methods”). Following 24 h of stimulation with T-MVs, CD34+ cells formed tubular structures (Supplementary Fig. 2A, B). Formation of branching networks in HUVECs and tubules in CD34+ cells were not prevented by antibodies against VEGF receptor 2 (KDR) nor Bevacizumab (in clinical use as an antiangiogenic drug) (Supplementary Fig. 2C, D), indicating that VEGF receptor 2 was not involved. Studies in retinas of 6-day-old (P6) mice 4 days after injection with T-MVs from different patients or C-MVs (6 μg) showed that T-MVs induce extensive formation of vascular sprouts positive for isolectin B4 (Fig. 2D). Only incomplete angiogenesis and disorganized cell accumulations were seen in C-MVs treated mice (Fig. 2D).

### Identification of the protein content of microvesicles

**Fig. 3:**
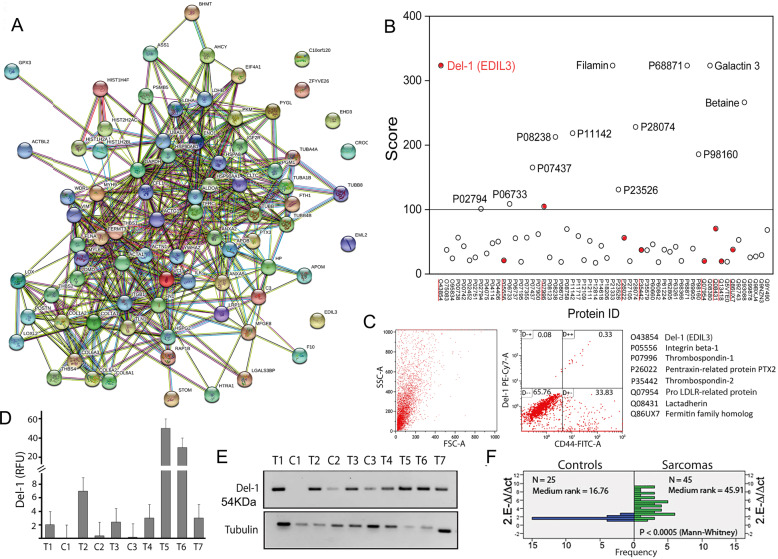
Mass spectrometry of protein content of T-MVs. **A** String analysis of all proteins present in T-MVs from sarcoma patients. **B** Plot reporting proteomic scores of 56 common proteins identified by three peptides by mass spectrometry and their identity code (ID). Proteins known to be involved in neoangiogenesis are underlined in red and identified below. **C** Fluorescence sorting of T-MVs unstained and stained with Del-1 and CD44 antibodies. The percent of Del-1 positive T-MVs is indicated. **D** Relative expression level of Del-1 protein in different control microvesicles (C1–C3) and tumor microvesicles (T1–T7) normalized for tubulin. The quantification was performed on three different western blots by Image J. **E** Western blots of protein extracts (30 µg) from individual MVs. Lanes C1–C3; proteins from three different control microvesicles from healthy subjects. Lanes T1–T7; proteins from MVs of seven different tumor patients. Tubulin was used as load control. **F** Expression levels of Del-1 mRNAs in giant cell tumor of bone sarcomas (*n* = 45) and bone cyst controls (*n* = 25) quantified by RT-qPCR (*n* = 3). Data are being reported as 2^-^^delta/delta ct^ versus frequency. Significance was calculated by non-parametric Mann–Whitney *T*-test.

To identify activators of the neoangiogenic process, proteins from T-MVs (plasma *n* = 3), from media of biopsy (*n* = 1) showing proangiogenic activity, and C-MVs from a healthy individual were analyzed by mass spectrometry. A shotgun proteomics approach indicated that T-MVs from different patients have very different content (Supplementary Table S2). Nevertheless, the comparison of proteins present in plasma T-MVs and cultured media T-MVs from biopsy of the same patient showed 56 common proteins present in duplicate determinations and identified by three peptides (Table S2 and Fig. 3A, B). String analysis of selected proteins identified some previously implicated in vessels formation, e.g., Del-1 integrin-binding protein. Given that Del-1 had one of the highest scores among angiogenic proteins (Fig. 3B) we confirmed its expression by FACS (Fig. 3C), and in plasma T-MVs by western blots (Fig. 3D). Moreover, Del-1 mRNA was expressed in 60% of GCTB biopsies (*n* = 45) at two- to fourfold higher level than in bone cysts (benign lesions) (*n* = 25) (Fig. 3E) and reported in several tumor tissues in Atlas Tumor Genomics Consortium bank (ATGC) (Supplementary Fig. 3A).

### Proangiogenic pathway activated by tumor microvesicles

To identify the early events of the present proangiogenic mechanism in target cells, we used a proteomic analysis comparing proteins from human endothelial cells stimulated with one T-MVs preparation (because all of them showed similar angiogenic result) with unstimulated cells. We did not include C-MVs stimuli because, although C-MVs contained small amounts of Del-1, they did not induce branches in HUVECs even at the highest concentration (Supplementary Fig. 1), and because a Del-inhibitor did not prevent angiogenesis by T-MVs (data not shown), suggesting that Del-1 is only one component of the present mechanism. Differential proteomic analysis comparing stimulated with unstimulated cells selected 90 differentially expressed proteins, 48 upregulated by up to 1.5-fold, and 42 downregulated (Supplementary Table S3). GO functional analysis using various bioinformatic tools (Cytoscape, String, and Reactome see “Material and methods”) showed that the calcium pathway and vesicle-mediated transport networks were the most upregulated (Supplementary Fig. 3B, C).

### Tumor microvesicles activate cytosolic calcium and mitochondria

**Fig. 4:**
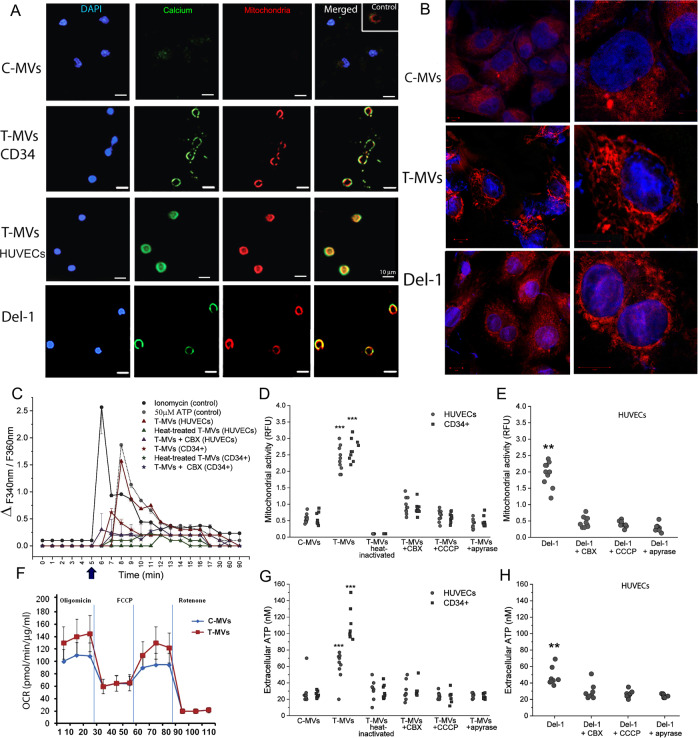
T-MVs increase cytosolic calcium, mitochondrial activity, and ATP release. **A** Representative confocal images of HUVECs and CD34+ cells stimulated by 6 μg/ml C-MVs or T-MVs, or by 1μM Del-1. After 10 min of stimulation, cells were stained for nuclei, mitochondria (Mitotracker red), and calcium (Fluo4-2AM green). Scale bar = 10 μm. **B** Image of HUVECs mitochondria stained with red mitotracker after 10 min of stimulation with C-MVs, T-MVs, or Del-1, showing progressive accumulation of mitochondria on the cell membrane and changes in their morphology. Scale bar = 10 μm objective ×40 and right zoom (×2). **C** Representative time-course of T-MVs induced changes in intracellular Ca^2+^, expressed as differential fluorescence (between C-MVs and stimuli) measured as ratio (F340/F380). HUVECs and CD34+ cells were incubated with 30 ng/ml Ionomycin or ATP 50 μM (positive controls), or stimulated with 6 μg/ml of T-MVs, heat-inactivated T-MVs) in the absence or presence of 50 μM CBX, a specific mitochondrial inhibitor. After 5 min of stimulation in Ca^2+^-free solution, 2.5 mM Calcium was added (indicated by arrow). Nonspecific Ca^2+^ entry was subtracted. Data are mean ± SEM of seven independent experiments per stimulus. **D** Mitochondrial activity (determined as averages of relative fluorescence intensity of MitoTracker (CM*-*H2XRos) in HUVECs and CD34+ cells stimulated for 5 min with C-MVs, T-MVs, heat-inactivated T-MVs, or T-MVs with inhibitors CBX (50 μM), CCCP (10 μM), or apyrase (20 U/ml). **E** Mitochondria relative fluorescence unit in HUVECs stimulated with Del-1 without or with CBX, CCCP, or apyrase. Data are mean ± SEM of three independent experiments. Significances were calculated by one-way ANOVA. **P < 0.05; ***P < 0.001 vs. all other groups. **F** Seahorse profile for oxygen-consuming rate (OCR) (normalized for concentration of protein reported as μg/ml) of HUVECs stimulated by T-MVs or C-MVs for 1 min. For a full description of the assay and its use to assess mitochondrial respiration see Supplementary Fig. 6. **G** Extracellular ATP in HUVECs and CD34+ cells stimulated for 1 min with C-MVs, T-MVs, heat-inactivated T-MVs, or T-MVs with inhibitors CBX (50 μM), CCCP (10 μM), or apyrase (20 U/ml). **H** Extracellular ATP in HUVECs stimulated with Del-1 without or with CBX, CCCP, or apyrase (the inhibitors were added 30 min before to start the test). Data are mean ± SEM of three independent experiments. Significances were calculated by one-way ANOVA. **P < 0.05; ***P < 0.001 vs. all other groups.

To confirm the involvement of calcium in this mechanism, we dosed cytosolic calcium by Fluo AM. Confocal images of cells stimulated with T-MVs showed an increase of intracellular calcium and mitochondrial staining compared to C-MVs cells (Fig. 4A, B). Fluorescence dosage indicated that intracellular cytosolic calcium (Ca^2+^) peaked in 1 min and is sustained for several minutes (Fig. 4C). Mitochondrial activity, oxygen-consuming rate, and extracellular ATP increased similarly (Fig. 4D–G). The mitochondrial inhibitor (CBX) reduced intracellular calcium accumulation, mitochondrial activity measured as potential membrane by MitoCMXRos, and ATP release (Fig. 4C, D), whereas uncoupling oxidative phosphorylation with carbonyl cyanide m-chlorophenyl hydrazine (CCCP) or blocking ATP release with apyrase and pannexin1 (inhibitors of purinergic receptor 7) reduced extracellular ATP and mitochondrial activity (Fig. 4D, E). This suggests that ATP originated from mitochondrial activity promoted by intracellular cytosolic Ca^2+^ accumulation. The addition of ionophore, a releaser of intracellular cytosolic Ca^2+^, did not induce mitochondrial activation or tubule formation (data not shown), indicating that other transduction molecules are involved. Similar results were also obtained with recombinant Del-1 protein (Fig. 4E, H).

### Intracellular signaling triggered by tumor MVs and the role of P2XR4

**Fig. 5:**
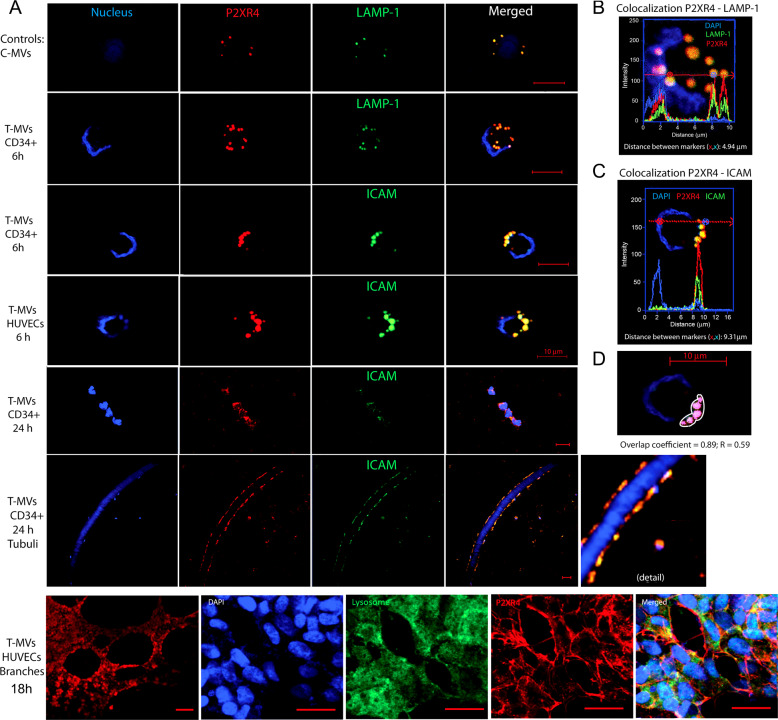
T-MVs increase P2XR4 expression. **A** Representative confocal fluorescence images using specific antibodies to show localization of P2XR4 (red), LAMP-1 (green) or the membrane marker ICAM (green), and nuclei (DAPI, blue) in both HUVECs and CD34 incubated for different times with C-MVs or T-MVs, as indicated. In both cell types, P2XR4 colocalizing with LAMP-1 then shifts toward the cell membrane, as indicated by P2XR4 colocalization with ICAM-1 after 6 h (scale bar = 10 μm). After 24 h CD34 cells appear elongated and aligned in a tubule-like structure positive for both P2XR4 and ICAM-1 (scale bar 10 μm). At 18 h HUVECs incubated with T-MVs on matrigel formed branches (scale bar 100 μm); nuclei stained with DAPI (blue) lysosome (green) P2XR4- (red) and ovelay. Scale bars = 10 μm. **B** Higher magnification of CD34+ cell merged image after 6 h incubation with T-MVs showing P2XR4 and LAMP-1 fluorescence intensity histogram along the cell plane indicated by the red arrow. **C** Higher magnification of HUVEC cell merged image after 6 h incubation with T-MVs showing colocalization between P2XR4 and ICAM-1 fluorescence intensity peaks almost exclusively on the cell membrane, at distance from the nucleus (DAPI blue). **D** Detail of overlap fluorescence staining (red P2XR4 and green ICAM). In the outlined area of this cell, Pearson’s overlap coefficient was 0.89 (*R* = 0.59). Data are mean ± SD of six experiments and a total of 55 cells.

To investigate the intracellular signaling after the calcium increase, proteomic analysis was performed on cells stimulated for 24 h with T-MVs, compared to unstimulated cells. Statistical analysis of mass spectrometry data identified 480 proteins by three different peptides in duplicate experiments, of which 356 were up- and 134 downregulated (Supplementary Table S4 and Supplementary Fig. 4A, B). Functional analyses using different bioinformatic tools showed that most overexpressed proteins gathered within two main pathways, vesicle-mediated transport, and energy metabolism (Supplementary Fig. 4B). Among the group of proteins selected by Heatmap (Supplementary Fig. 4A and Supplementary Table 4), we focused further investigation on purinergic X recetor 4 (P2XR4), because this ATP-gated channel promotes Ca^2+^ influx and T-cell motility [24]. P2XR4 was the unique receptor of the purinergic family selectively increased following T-MVs stimulation. In our experimental setting P2XR4 expression in non-stimulated cells or C-MVs was very low (Fig. 5C). After 6 h of stimulation by T-MVs, the dense cluster of P2XR4 staining colocalized with lysosome-associated membrane glycoprotein 1 (LAMP-1) an integral lysosome membrane protein. Moreover, in some cells P2XR4 protein was also colocalized with ICAM-1 membrane protein, mostly on one side of the cell (Fig. 5A, B), with a Pearson coefficient of overlap of 89% (*R* = 0.59) (Fig. 5D) and on the surface of tubules (Fig. 5A). In HUVECs stimulated with T-MVs, the branches formed showed an intense lysosome staining and P2XR4 delinated the cell membrane (Fig. 5A lower panel). The increase and surface presentation of P2XR4 were also induced by recombinant Del-1 protein (Supplementary Fig. 4C) and other proangiogenic stimuli, such as chemokines CXCL12 (SDF-1) and CCL5 detected in our proteomic analysis (Supplementary Fig. 5). Moreover, no evidence of autophagic mechanism increase was revealed in T-MVs stimulated cells compared to controls (Supplementary Fig. 6A).

### P2XR4 mediates vesicular trafficking and endothelial cell migration

**Fig. 6:**
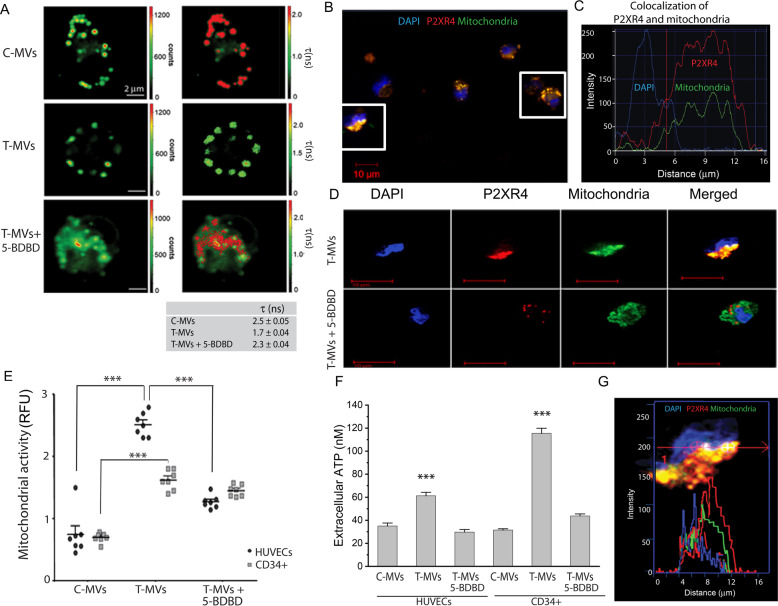
Vesicular trafficking of P2XR4 affects membrane viscosity. **A** Fluorescence Lifetime Imaging Microscopy (FLIM) evaluating fluorescence intensity (left images) and average fluorescence lifetime (right images) in single-cell stained with 5 μM BODIPY FL C12 for 20 min following specific stimuli, as indicated. Scale bars = 2 µm. Mean of *τ* values (in nanoseconds) calculated from the green to red pseudo-color scales directly relate to membrane viscosity, and are reported as mean ± SEM of three independent measurements of *n* = 20 cells for each plate. **B** Representative images of HUVECs stimulated with T-MVs, overlay of mitochondria (green) and P2XR4 antibody (red). Scale bar = 10 μm (20× objective). **C** Example of typical fluorescence intensity histogram showing DAPI (nuclei, blue) P2XR4 (red) and mitochondria (green) staining. **D** Representative images of HUVEC cell stimulated with T-MVs, or T-MVs with the P2XR4 inhibitor 5-BDBD (5 μM), stained for mitochondria (green) and P2XR4 antibody (red) and merged. Scale bar = 10 μm (60× objective). **E** Relative fluorescence intensity of MitoXRos staining (CM*-*H2XRos) in HUVECs and CD34+ cells treated with C-MVs, T-MVs, or T-MVs + 5-BDBD. **F** Extracellular luciferase ATP dosage in CD34 cells and HUVECs after 1 min of stimulation with C-MVs or T-MVs without or with 5-BDBD (5 μM). Data are mean ± SEM of three independent experiments. Significances were calculated by one-way ANOVA. ***P < 0.001 vs. control and T-MVs + 5-BDBD. **G** Fluorescence intensity peaks of P2XR4 and Mitochondria staining along the diameter of HUVEC cell stimulated with T-MVs (merged panel **D**).

To confirm lysosome and P2XR4 trafficking onto the cell membrane, we used FLIM. Because the fusion of lysosomes (intracellular membranes) with the extracellular membrane alters the viscosity of the latter [22], we measured the fluorescence lifetime of the molecular rotor 4,4-difluoro-5,7-dimethyl-4-bora-3a,4a-diaza-s-indacene-3-dodecanoic acid (BODIPY FL C12) [25]. Cell stimulation by T-MVs reduced BODIPY lifetime to 1.7 ns compared to 2.5 ns of C-MVs, indicating an increase of membrane permeability (Fig. 6A). In order to investigate the link between T-MVs, calcium, and P2XR4 we inhibited P2XR4 by chemical and genetic approach. We assessed that 5-BDBD antagonist reduces the viability of T-MVs stimulated cells in a dose-dependent manner (Supplementary Fig. 6B, C). Oxygen consumption rate, as measure of mitochondrial function, decreased in a dose-dependent manner, early after addition of 5-BDBD, as did mitochondrial ATP production (Supplementary Fig. 6D, E). 5-BDBD at dose of 5 μM was effective to reduce HUVECs membrane viscosity, a measure of membrane dynamic trafficking (Fig. 6A). 5-BDBD (5 μM) prevented mitochondrial localization to one cell pole (Fig. 6B-D) reduced mitochondrial activity, and extracellular ATP release (Fig. 6E, F). Moreover, 5-BDBD affected two essential steps of neangiogenesis-motility and branch formation- in a dose-dependent manner (Supplementary Fig. 6F–H). Pharmacological inhibition of P2XR4 also reduced Del-1 activity in stimulated HUVECs (Supplementary Fig. 7). Genetic ablation of P2XR4 by siRNA trasfection attenuated cell proliferation by 3%, measured as the amount of Ki67-positive cells, compared to scramble siRNA trasfected cells (Supplementary Fig 8A–C). siP2XR4-RNA influenced cell migration in the wound-healing assay and branch formation. As indicated in supplementary Fig. 8 control HUVECs and those transfected with siRNA scramble closed 80% of the wound in 12 h, whereas cells transfected with siRNA of P2XR4 inefficiently sealed the wound over the same time frame (Supplementary Fig. 8D, E). In addition, siP2XR4-RNA transfection reduced significantly the capability of HUVECs to form branches on matrigel (Supplementary Fig 8G, H). Therefore, these data demonstrated that P2XR4 is required for HUVECs tube formation, proliferation, migration, and mitochondrial energy production (Supplementary Fig. 8).

### Inhibition of P2XR4 reduces tumor growth and angiogenesis in vivo

**Fig. 7:**
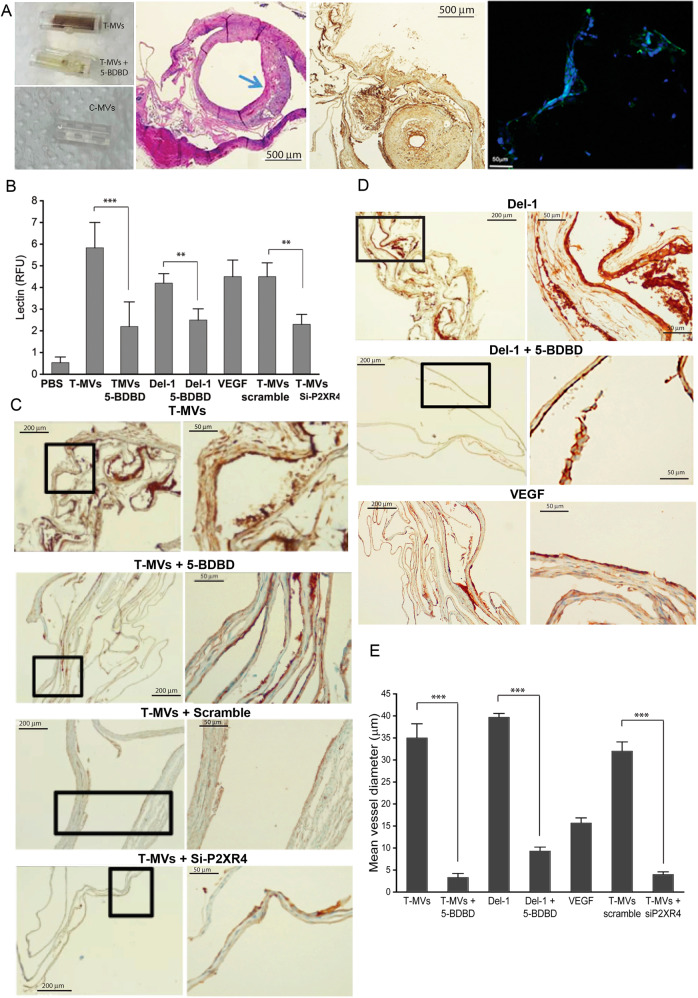
Inhibition of P2XR4 activity reduces neoangiogenesis under non-pathological conditions. **A** Left: representative images of angioreactors filled with T-MVs, T-MVs + 5-BDBD or C-MVs explanted after 14 days. Middle: representative image of larger vessels developed within angioreactor, stained with H&E (left) or immunostained with CD31 antibody (right). Scale bars = 500 μm. The arrow indicates intimal thickening. Right: Representative fluorescence image of vessels removed from angioreactors filled with PHK23-stained T-MVs. Nuclei are stained with DAPI (blue). The green color indicates PHK23-stained MVs in the cytoplasm of vascular cells. Scale bar = 10 μm. **B** Quantitative evaluation of FITC lectin (read at 550 nm) of vessels extracted from angioreactors. Data are mean of four angioreactors per treatment group ± SD; ***P < 0.001, **P < 0.05 vs. the respective groups containing the P2XR4 antagonist (5-BDBD) or scramble v.s. siP2XR4-RNA. Significance was calculated using Student’s two-tailed t-test. **C** Representative micrographs of CD31-stained vessels developed in angioreactor filled with T-MVs with or without 5-BDBD, T-MVs + scramble, or T-MVs + siP2XR4. Low magnification images on the left (scale bars = 200 μm) and high magnification images on the right (scale bars = 50 μm). **D** Similar low- and high-magnification images of vessels filled with Del-1 without or with 5-BDBD or with VEGF. **E** Quantitative assessment of vessel diameters in angioreactors. Data are mean ± SD. ***P < 0.001 by ANOVA. The image was analyzed by Image J.

**Fig. 8:**
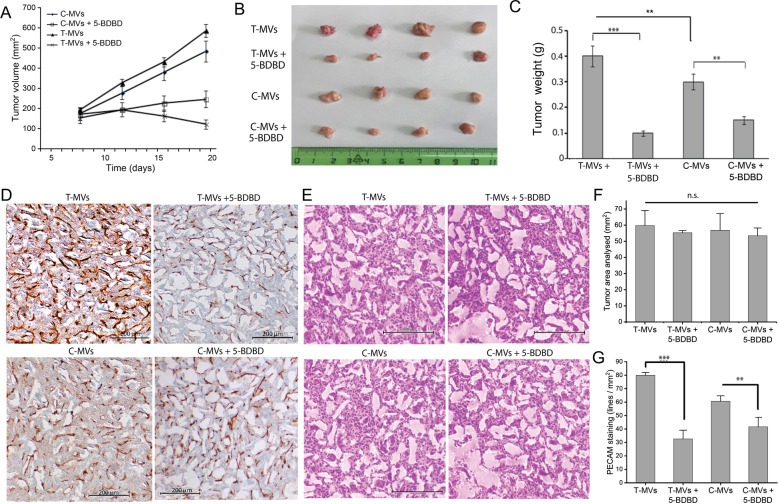
Inhibition of P2XR4 reduces in vivo tumor angiogenesis in athymic mice. **A** Tumor growth in mouse xenographs over 3 weeks by three treatments per week with C-MVs or T-MVs (70 μg/ml of mouse blood) with or without 5 mg/kg of 5-BDBD. **B**, **C** Representative images of excised tumors and tumor weights at day 21. Data are mean of four tumors per treatment group ± SD; **P < 0.05 vs. C-MVs. ***P < 0.001 vs. mice treated with T-MVs + 5-BDBD or C-MVs (two-tailed Student’s *t*-test. **D** Tumor sections from mice treated with T-MVs, T-MVs + 5-BDBD, C-MVs, or C-MVs + 5-BDBD, immunostained for CD31. **E** Serial tumor sections stained with H&E. Scale bars = 100 μm. **F** Sections of tumor area from different treatment group (as indicated) stained H&E. **G** Quantitation of PECAM (CD31-stained vessels per mm^2^ of tumor area from mice treated with C-MVs (*n* = 9 slides), C-MVs + 5-BDBD (*n* = 8 slides), T-MVs (*n* = 12 slides) or T-MVs + 5-BDBD (*n* = 10 slides). Statistical significance was calculated using the two-tailed *t*-test. **P < 0.05 and ***P < 0.001 vs. the group receiving the same MVs but no P2XR4 inhibitor.

To investigate the in vivo relevance of the present mechanism, we examined whether T-MVs and Del-1 protein can promote vessel formation in angioreactors under non-pathological conditions [26]. For this purpose, nude/nude mice were implanted angioreactors filled with matrigel containing VEGF, T-MVs, Del-1, C-MVs, all w/wo 5 μM 5-BDBD, or with scramble or siP2XR4-RNA, and injected intraperitoneally with the same agents for 14 days (see “Material and methods”). Angioreactors from T-MVs treated mice showed numerous vessels, compared to mice treated with T-MVs + 5-BDBD, whereas no vessels were present in C-MVs angioreactors (Fig. 7A). Histological examination indicated a higher number and larger diameters of CD31+ vessels in mice treated with T-MVs, compared to T-MVs + 5-BDBD and with T-MVs+ scramble, compared to siP2XR4 (Fig. 7B, C). Similar results were obtained in angioreactors filled with Del-1, compared to Del-1 + 5-BDBD (Fig. 7D). Fluorescence lectin quantification of vessels (extracted from angioreactors) indicated a threefold decrease of vascularization in 5-BDBD-treated mice compared to T-MVs and twofold decrease in siP2XR4 mice compared to scramble (Fig. 7E). None of the treatments affected the number of circulating CD34+ cells (data not shown). To confirm the above findings under pathological conditions, xenograft mouse tumor models were intraperitoneally injected with T-MVs or C-MVs, and 5-BDBD. Explanted tumors from mice treated with T-MVs + 5-BDBD or C-MVs + 5-BDBD were significantly smaller (i.e., had lower weight) than those of mice receiving only T-MVs or control C-MVs (*P* < 0.001) (Fig. 8A–C). A significant difference was also observed between T-MVs and C-MVs treatment in final tumor size and weight (*P* < 0.05) (Fig. 8A–C). Histological examination of size-matched tumor sections revealed 50% fewer CD31+ cells per area in tumors treated with T-MVs + 5-BDBD, compared with T-MVs alone, whereas tumor vascularization was significant higher in T-MVs then C-MVs. (Fig. 8D–G). In addition, CD31+ positivity was greater in C-MVs treated tumors than in those of the C-MVs + 5-BDBD group, suggesting that vessels developing spontaneously during tumor growth were reduced by 5-BDBD treatment.

## Discussion

The present study shows that T-MVs from sarcoma patients can activate neangiogenesis in non-pathological conditions and tumor environments, and that this is reduced by pharmacologic and genetic inhibition of P2XR4. Purinergic signaling has been shown to mediate a variety of cancer-related processes in the tumor microenvironment [27, 28]. P2XR4 was previously reported to control vascular tone and remodeling in response to hypoxia [29] pulmonary hypertension [30], inflammation and pain in dorsal root neuron ganglions [31], cell motility in immune response [24], and mediated thymosin b-4 HUVEC motility [32]. We now provide evidence for the role of P2XR4 in neoangiogenesis promoting tumor growth. The mechanism here reported involves an increase of cytosolic calcium and mitochondrial activity, sustained by Ca^2+^ influx and ATP consumption via the P2XR4 receptor.

The role of mitochondrial energy in cell migration during angiogenesis is well established, but it is not definitively established whether receptor pathways are involved [33, 34]. Our differential proteomic analysis indicated that P2XR4 is the sole purinergic receptor increased by tumor MVs stimulation of endothelial cells. T-MVs stimulation induces the translocation and clustering of lysosomal P2XR4 on the cell membrane, where it sets in motion a feed-forward mechanism to further increase intracellular calcium, mitochondrial activity, and ATP production. Dissecting this mechanism by uncoupling the mitochondrial respiratory chain and inhibiting P2XR4 receptor activity provides evidence that both are necessary to promote cell migration and formation of branching tubular networks.

Given that the composition of patient T-MVs varies greatly, we tried to analyzed their protein content. Our proteomic analysis identified Del-1 protein and confirmed its increased presence in MVs isolated from plasma tumor patients, compared to controls from healthy subjects, as well as in many tumor biopsies of GCTB patients. The involvement of Del-1 in angiogenesis has long been assumed [35–38]. Del-1 regulates leukocyte recruitment, promotes resolution of inflammation by mediating phagocytosis of apoptotic neutrophils [39], and bone osteogenesis [40]. In contrast, the metabolic mechanism triggered by T-MVs containing Del-1 in tumor angiogenesis was not known. We showed that Del-1 and T-MVs positive to Del-1 together with at least two established proangiogenic factors, (CCL5 and CXCL12) polarizes P2XR4 receptors on cell membranes [41]. This may also mean that P2XR4 is involved in cell motility in general. In vivo experiments confirmed that blocking P2XR4 activity reduces tumor vascularization promoted by T-MVs but also, to a lesser extent, by C-MVs. Results indicate that P2XR4 is a key downstream effector of multiple proangiogenic factors promoting tumor vascularization, although we cannot exclude that P2XR4 could be also involved in sarcoma cell growth [42]. Collectively, the present findings provide proof-in-principle that P2XR4 is a key player in a proangiogenic mechanism that connects mitochondrial activity and endothelial cell motility and promotes tumor vascularization and growth. Moreover, we provide evidence that P2XR4 is a potential target to modulate neoangiogenesis.

## Supplementary information

Supplementary methods

Supplementary legends to figures and tables

Supplementary Figure 1

Supplementary FIgure 2

Supplementary Figure 3

Supplementary Figure 4

Supplementary Figure 5

Supplementary FIgure 6

Supplementary FIgure 7

Supplementary FIgure 8

Supplementary Table 1

Supplementary Table 2

Supplementary Tables 3 and 4

Data set 1

## Data Availability

The data that support the findings of this study are available from the corresponding author upon reasonable request
