# Supplementary material for: Lysosome purinergic receptor P2X4 regulates neoangiogenesis induced by microvesicles from sarcoma patients
Source: Cell Death Dis. 2021 Aug 17;12(9):797. doi: 10.1038/s41419-021-04069-w (PMC8371002; doi:10.1038/s41419-021-04069-w)
Supplement: Supplementary file 1 — Supplementary methods [file 41419_2021_4069_MOESM1_ESM.docx]

**Supplementary Methods**

**Microtubuli formation**

Tube formation was assessed using 15 well μ-Slide Angiogenesis ibidi Treat plates (Ibidi) and following the instructions of the Application Note. Briefly, 10μl Matrigel was added to each well. The μ-Slide plate was immediately placed in the incubator to allow gel polymerization for 30 min. Next, 1x10^4^ HUVECs suspended in 50 μl of EGM-2 were added to each well and incubated at 37°C in 5% CO_2_; 18 h later, pictures were taken using a phase contrast inverted microscope Olympus IX-71 (10x objective). To analyze siRNA-mediated inhibition, cells were transfected 24 h before experiments. To analyze inhibition by 5-BDBD, the drug or vehicle (DMSO) was added to wells after cell seeding.

**Endothelial cell migration**

HUVEC migration in vitro was evaluated by wound-healing assay. Briefly, HUVECs were seeded in 6- well plates and cultured until reaching 90–95% confluence. Then, using a sterile 2 – 200 μl pipette tip, a scratch was applied. After washing with PBS 1, 1 ml of EGM-2 was added to each well. For siRNA experiments, HUVECs were transfected 48 h before scratching at 70% confluence. To analyze inhibition by 5-BDBD, the drug or vehicle (DMSO) was added after scratching. Pictures were immediately taken by an inverted phase contrast microscope Olympus IX71 (10 objective), and 12 h after scratching. The cell-free area in the wound was measured using ImageJ (NIH, Bethesda, MD, United States).

**Cell proliferation**

Viability of HUVECs was measured by MTT assay (Sigma). Briefly, HUVECs were seeded at 2×10^3^ cells/well in 96-well plates in medium containing T-MV or CMVs (6 μg/ml) for 1h. 5-BDBD or vehicle were then added at different dosage. The treatment medium in each well was replaced with MTT labeling reagent (final concentration 0.5 mg/ml). Microplates were incubated for 4 h in a humidified atmosphere (37°C, 5% CO_2_). One hundred μl of the solubilization solution were added to each well, and plates were kept overnight in the incubator. Absorbance was measured using a microplate (ELISA) reader, using absorbance 550 and 600 nm according to the filters available for the ELISA reader, TECAN INFINITY 2000. For siRNA experiments HUVECs were seeded on coverslips, and cell transfection was done 48 h before the assay. The cells were fixed with formalin, permeabilized with PBS 0.5% Triton X-100, and blocked with PBS 1 containing 1% bovine serum albumin and 0.5% TWEEN 20. HUVECs were then incubated with a mouse anti-Ki67 antibody for 2 h at room temperature. Coverslips were then incubated for 45 min in the dark with Goat anti-Mouse Alexa FluorR 488 (1:200 dilution). HUVECs were incubated with DAPI diluted in PBS for 5 min to visualize the nucleus. The coverslips were mounted using Vectashield. Cells were photographed using an Zeiss microscope (10 x objective). To analyze apoptotic cells, we used propidium iodide (2mg/mL).

**Seahorse experiments**

Mitochondrial respiratory capacity was detected by the Seahorse XF Cell Mito Stress Test Kit (103015-100, Agilent, Delaware, USA) which directly measures the oxygen consumption rate (OCR), using an Agilent Seahorse Xfe96 Extracellular Flux Analyzer (Agilent, Delaware, USA). Cells (10^4^/well) were seeded onto Seahorse XF cell culture 96-well microporous plates (103729-100, Agilent, Delaware, USA). Oligomycin A, FCCP, and antimycin A at final concentrations of 1.5, 1, and 0.5μM, respectively, were then added to each well, and the OCR measured according to the manufacturer’s instructions. Normalization was performed with total protein extract.

**Transfection siRNAs**

P2X4R mRNA and protein expression in HUVECs was silenced by siRNATrilencer-27 transfection for 48 h with 5 nM P2X4R siRNA or 5 nM Scrambled siRNA ([SR303323](about:blank) OriGene Technologies; Rockville, MD, USA), along with jetPRIME transfection reagent (Polypus transfection, Bioparc, France) were used to transfect cells to obtain sufficient knockdown. Briefly, cells were seeded at a density of 1×10^5^ in T25 flasks. The transfection reaction mixture contained 10 nM P2X4R siRNA or scrambled siRNA, 400 µl jetPRIME buffer, and 8 µl jetPRIME transfection reagent was incubated together to form duplexes (according to manufacturer’s recommendations). Cells were transfected for 24 h, following a media change for 48 h, and used to evaluate downstream signaling studies.

**Primers used for real-time PCR were:**

| Del-1 | 5′-TCGAAGACATTGCACTTTGC-3’ | 5′-ACCCAGAGGCTCAGAACAAC-3′), |
| --- | --- | --- |
| β-actin | 5′-TTGCCGACAGGATGCAGAA-3′ | 5′-GCCGATCCACACGGAGTACT-3′). |
| P2XR4 | 5’-GCAGAAAACTTCACCCTCTTGG-3 | 5’-AGGTAGGAGGTGGTAATGTTGG-3’ |

**Special reagents**

The following molecules were added to the cell medium: 5-BDBD (5 μM in vitro, 4.25 mg/kg, i.p. in vivo; cas 768404-03-1 Sigma-Aldrich); recombinant human Del-1 protein (1 μM in vitro; 2,3 mg/kg i.p. in vivo; CF 6046-ED-050 R&D Systems); recombinant CXCL12, (10 ng/ml; 350-NS-010 R&D System), mitochondrial inhibitor Rotenone (CBX); (50 μM; CAS 83-79-4), PANX1 inhibitor (10 μM; CAS.[955091-53-9](about:blank)); Adenosine 5′-triphosphate (ATP) disodium salt hydrate (10 μM CAS 68807-90-9); CCCP (5 µM, CAS 555-60-2) and Apyrase (20 U/ml; CAS 68807-90-9) (all from Sigma-Aldrich); recombinant fibronectin (10 ng/ml 1030-FN-05M); recombinant human VEGF 165 (10 ng/ml in vitro or 100 ng/ml in angioreactors and in vivo retina; 293-VE-050 from R&D Systems); Ionomycin (I24222, Thermo Fisher). For cytometric analysis the following primary antibodies were used: mouse anti CD44-FITC (BD Biosciences), mouse anti human CD117 V450 (BD Biosciences), mouse anti human CD90 PE-Vio770 (Miltenyi Biotech), mouse CD63 FITC (Antibodies Online), mouse CD9 PE CY5 (Life Span), and mouse DEL-1 PE (bs-7029R-PE-Cy5, Bioss). MVs were defined as having a diameter < 1 μm and being positive for cell-specific markers and negative for 7-aminoactinomycin D (7-AAD) (Invitrogen).
