# Supplementary material for: Lysosome purinergic receptor P2X4 regulates neoangiogenesis induced by microvesicles from sarcoma patients
Source: Cell Death Dis. 2021 Aug 17;12(9):797. doi: 10.1038/s41419-021-04069-w (PMC8371002; doi:10.1038/s41419-021-04069-w)
Supplement: Supplementary file 2 — Supplementary legends to figures and tables [file 41419_2021_4069_MOESM2_ESM.docx]

**Lysosome purinergic receptor P2X4 regulates neoangiogenesis induced by microvesicles from sarcoma patients**

Wulf Palinski,^1 #*^ Maria Monti,^2#^ Rosa Camerlingo,^3^ Ilaria Iacobucci,^2^ Serena Bocella,^4^ Federica Pinto,^4^ Clara Iannuzzi,^5^ Gelsomina Mansueto,^6,7^ Sara Pignatiello,^6^ Flavio Fazioli,^8^ Michele Gallo,^8^ Laura Marra,^3^ Flora Cozzolino,^2^ Annarosaria De Chiara,^9^ Piero Pucci,^2^ Antonio Bilancio,^5^ Filomena de Nigris^5*^

1 Department of Medicine, University of California San Diego, La Jolla, California, USA

2 Department of Chemical Sciences, University of Napoli Federico II and CEINGE

Advanced Biotechnologies, Naples, Italy

3 Department of Cell Biology and Biotherapy Research, Istituto Nazionale Tumori IRCCS - Fondazione G. Pascale, Naples, Italy

4 Department of Experimental Medicine, University of Campania “Luigi Vanvitelli”, Naples, Italy

5 Department of Precision Medicine, University of Campania “Luigi Vanvitelli”, Naples, Italy

6 Department of Advanced Biomedical Sciences, University of Naples Federico II, Naples, Italy

7 Department of Advanced Medical and Surgical Sciences, University of Campania “Luigi Vanvitelli”,

8 Division of Skeletal Muscle Oncology Surgery, Istituto Nazionale Tumori IRCCS - Fondazione G. Pascale, Naples, Italy

9 Division of Anatomy, Istituto Nazionale Tumori IRCCS - Fondazione G. Pascale, Naples, Italy

* email: wpalinski@ucsd.edu ; filomena.denigris@unicampania.it

**Legends of Supplementary Figures and Tables**

**Suppl. Fig. 1 (Related to Figure 2). Dose response branch formation. a Representative image of HUVECs grown on Matrigel (μSlides, Ibidi) treated with different doses (100 ng, 1 μg, 5 μg/ml) of tumor microvesicles (T-MVs) from different patients, control microvesicles (C-MVs) from two healthy subjects, or heat inactivated T-MVs for 18 h. As a positive control, cells were incubated with VEGF (100 ng/ml, 1 μg/ml, or 10 μg/ml). Scale bar = 100 μm. b-f Quantitative comparison of the number of branches per visual field (n=4, analyzed with 10x objective) for each stimulus.** Data are results of 3 experiments. ***P < 0.001 vs. all other groups.

**Suppl. Fig. 2 (Related to Figure 2). a Upper panels**: Representative confocal immuno-fluorescence images of a tubule formed by CD34+ cells stimulated with T-MVs for 24 h and stained with CD31 (PECAM) or ICAM-specific antibodies, and control CD34+ cells. Lower panels: CD34+ cells stimulated with T-MVs for 24 h and stained with CD31 (PECAM) or ICAM-specific antibodies. Scale bars = 10μm. **b** Three-dimensional analysis of DAPI-stained tubule by confocal laser scanning microscopy showing distinct walls and a patent lumen. Colored lines indicate the planes of the orthographic projections. Scale bar = 50 μm. Lower panel: zoom of branching area (indicated by the frame). Scale bar = 20 μm. **c** Representative immunofluorescence images of tubules formed by 10^4^ CD34+ cells stimulated for 24 h with 6 μg/ml T-MVs alone or together with 10 μg/ml of a neutralizing antibody to VEGFR2 (KDR), and with 10 ng/ml VEGF cells alone or together with KDR. Images are merged DAPI (blue) and CD31 (green) staining. Scale bars = 20 μm. **d** Representative image of 1x10^4^ HUVECs stimulated by PHK23-labeled T-MVs alone or together with 10μg/ml of anti-KDR antibody or 10 μg/ml of Bevacizumab. Scale bars = 100 μm.

**Suppl. Fig. 3 (Related to Figure 3). Proteomic analysis of tumor microvesicles. a Del-1 mRNA expression in several tumors from cancer genomic ATLAS (ATGC) data base**. **b** ClueGO pathway analysis of proteins selectively increased up to 1.5 fold in cells stimulated by T-MVs after 30 min vs. controls (Table S3) and their Benjamini Hochberg significance. **c** Reactome diagram of proteins increased by up to 1.5 fold in T-MVs-stimulated cells after 30 min. The color of the legend indicates the statistical significance of each pathway.

**Suppl. Fig. 4** (**Related to Figure 4).** **Proteomic analysis of cells stimulated for 24 h with tumor microvesicles**. **a** Heat-map showing data reproducibility between replicates within each condition (treatment vs. control at 24 h). Based on normalized protein values, two big clusters are identified that show opposite trends. Cluster 1, composed of 356 proteins, was increased in T-MVs stimulated cells, whereas cluster 2, composed of 134 proteins, was decreased in T-MVs. Raw data were normalized using the Z-score method and hierarchical clustering by considering Euclidean distance for both groups (treatment/control) and protein levels. Statistical analyses were performed using R software. Hierarchical clustering and heatmap were carried out using the heatmap function. All data are provided in Supplementary Table 4. **b** Reactome diagram of proteins increased in T-MVs stimulated cells vs. unstimulated control cells after 24 h. The color of the legend describes the statistical significance of each pathway. **c** Table showing highly significant proteins (P < 0.001) selectively expressed in human endothelial cells after 24h of treatment with T-MVs. **d** CD34+ cells stimulated for 6 h with recombinant Del-1 (1 μM) and unstimulated control stained with antibodies to P2XR4 and ICAM, as indicated. Scale bars = 10 μm.

**Suppl. Fig. 5 (Related to Figure 5). Purinergic X receptor 4.** Confocal images of single cells stimulated with Del-1, VEGF, CCL5 and SDF-1 for 24 h, stained with DAPI (blue) or an antibody specific for P2XR4 (red). Scale bars = 10 μm.

**Suppl. Fig. 6 (Related to Figure 6). Effects of different doses of 5-BDBD on proliferation, motility, mitochondrial activity and formation of branching networks. a Western blot immunostained with LC3-I and LC3II of protein extracts from HUVECs stimulated for 24 h with C-MVs (a) and T-MVs (b), together with PONCEAU indicating equal load (left). b Viability of HUVECs stimulated with C-MVs or T-MVs and treated with different doses of 5-BDBD for 24 h, assessed by MTT assay. Data are mean of 3 experiments. c Determination of the contribution of MVs and 5-BDBD to cell death assed by cell sorting. HUVECs were grown for 24 h with T-MVs or C-MVs in the absence or presence of 5 μM or 1 μM 5-BDBD. Dot plots show cell death assessed by propidium iodide staining. d Seahorse profile for oxygen consuming rate (OCR) of HUVECs stimulated by T-MVs (control) or treated for 1 min with different doses of 5-BDBD (0.5 μM, 1 μM, 5 μM). Treatment with 1.5 μM oligomycin, 0.5 μM FCCP, and 1.5 μM antimycin/rotenone** were used to asses mitochondrial respiration. Data were normalized by protein concentration. **e** ATP mitochondrial production measured as proton leak. Data are mean  ±  SEM. **P  < 0.05; ***P < 0.001. **f** Effects of 5-BDBD on motility of HUVECs. 10^5^ HUVECs were plated, grown to confluence, scratched, and stimulated with tumor microvesicles (T-MVs) or control microvesicles (C-MVs) alone or together with 5-BDBD (5 μM) or its vehicle. Scale bars = 100 μm. Wound closure rates were determined as the percent of the scratched areas closed by adjacent cells after 6 or 12 h, as indicated. The edges of the cell-free scratch area are indicated by tracer lines. Phase-contrast images. Scale bars = 100 μm. **g** Qantitative assessment of wound closure. Data represent three independent experiments. ***P  < 0.001. vs vehicle. **h** Left: Typical images of HUVECs grown on Matrigel and stimulated for 18 h with T-MVs in the absence or presence of 5-BDBD. T-MVs incubated with the vehicle of the inhibitor served as control. Right: Quantitative assessment of branch formation for two doses of 5-BDBD. Data are means ± S.E.M. ***P < 0.01 vs. 5-BDBD treatment.

**Suppl. Fig. 7 (Related to Figure 6).** **Fluorescence Lifetime Imaging Microscopy (FLIM and mitochondrial activity. a** Fluorescence Lifetime Imaging Microscopy (FLIM) in single vital cells stained with 5 μM BODIPY FL C12 for 20 min, following specific stimuli indicated. Scale bars = 2 µm. **b** Mean of τ values (in nanoseconds) calculated using the pseudo-color scales are directly related to membrane viscosity, and expressed as mean ± SEM of three independent measurements of n = 20 cells for each plate. **c** Intracellular calcium concentration in cells stimulated with Del-1 protein without or with 5-BDBD (5 μM) after 1 min. **d** Relative fluorescence units of mitochondrial activity (determined by MitoTracker CM*-*H2XRos) in cells stimulated without or with 5-BDBD for 5 min.e Extracellular ATP in cells stimulated without or with 5-BDBD for 5 min. Data are mean ± SEM of 3 independent experiments. Significances were calculated by one-way ANOVA ***P <0.001 vs. control or Del-1 + 5-BDBD.

**Suppl. Fig. 8 (Related to Figure 6)**. **siRNA-mediated inhibition of P2XR4 attenuates HUVEC microtubule formation, proliferation, and migration. a** Levels of P2XR4 mRNA expression (log fold change) in control HUVECs (n = 8) and in HUVECs transfected with scramble (n = 4) or siRNA P2XR4 (n = 5). ***P < 0.001. **b** Representative recordings of intracellular changes in Ca^2^, measured as fluorescence ratio (F340/F380) of control cells or cells transfected with scramble (n = 4) or siRNA P2XR4 (n = 5) after stimulation by T-MVs or C-MVs. Ionomycin (30 μM) or ATP (50 μM) were used as positive controls. The stimulation was conducted for 5 min in a Ca^2^-free solution. Then 2.5 mM Ca^2+^ was re-added (indicated by arrow) together with different stimuli, such as T-MVs or C-MVs. **c** Representative merged images of HUVECs transfected with scramble or siRNA P2XR4. Cells were stained with Ki67 (green) and DAPI (blue). Scale bars = 10 μm. The bar graph to the right shows the percentage of Ki67 (puncta) in HUVECs stimulated with T-MVs (controls) or transfected with siP2XR4. Data are normalized to scramble. **d** Phase-contrast imaging of a wound healing assay in HUVECs. The scratch assay was carried out with control cells or cells transfected with scramble siRNA or siRNA P2XR4 (siP2XR4). Images show the scratch area at t = 0 and 12 h. The percentage of wound closure was determined using Image J. Scale bars = 100 μm. The bar graph to the right shows quantitative results. **e** Phase-contrast images of control HUVECs, or HUVECs transfected with scramble or siRNA P2XR4, grown on Matrigel and stimulated with T-MVs for 18 h. The graph on the right shows the quantitative evaluation of the effect of P2XR4 silencing on the number of branches formed. Data in the bar graphs C-D were normalized to scramble and are mean ± SEM of 4 experiments. **P < 0.05 and ***P < 0.001.

**Table S2 (Excel file): Proteomic analysis of patient microvesicles. Sheet 1:** Proteins identified in MVs from plasma of patient 1 compared to MVs prepared from culture media of cells (peptides A) isolated from the same patient’s tumor biopsy (peptides B). Proteins were identified in mass spectrometric analysis by two peptides, and data reflect duplicate LFQ measurements with P < 0.05. **Sheet 2** Proteins identified in MVs from plasma of patient 2; **Sheet 3** Proteins identified in MVs from plasma of patient 3; **Sheet 4** Proteins identified in MVs from plasma of patient 4; **Sheet 5** Proteins identified in MVs from plasma of healthy donor (see extended Table 2) .

**Supplementary Table 3 (Excel file): Proteomic analysis of CD34+ cells stimulated with tumor microvesicles for 30 min.** Proteins selectively expressed or significantly increased in CD34+ cells incubated with tumor microvesicles, compared to non-stimulated controls, were determined by quantitative mass spectrometry. Proteins identified by at least three peptides showing significant differences in label free quantification (LFQ) (P < 0.05). Data represent three experiments with triplicate determinations. FC = fold change. CRT= control; STIM=stimuli; FC= fold change.

**Supplementary Table 4 (Excel file): Proteomic analysis of CD34+ cells incubated with tumor microvesicles for 24 hours.** Comparative quantitative proteomic analysis of cells incubated with angiogenic microvesicles and non-incubated controls indicated 490 proteins identified by 3 peptides that were significantly increased by label free quantification (LFQ). The first 93 proteins on the list were unique to stimulated cells. Data represent duplicate determinations. CRT= control; STIM=stimuli; FC= fold change.
