# Supplementary figures and images for: Lysosome purinergic receptor P2X4 regulates neoangiogenesis induced by microvesicles from sarcoma patients
Source: Cell Death Dis. 2021 Aug 17;12(9):797. doi: 10.1038/s41419-021-04069-w (PMC8371002; doi:10.1038/s41419-021-04069-w)

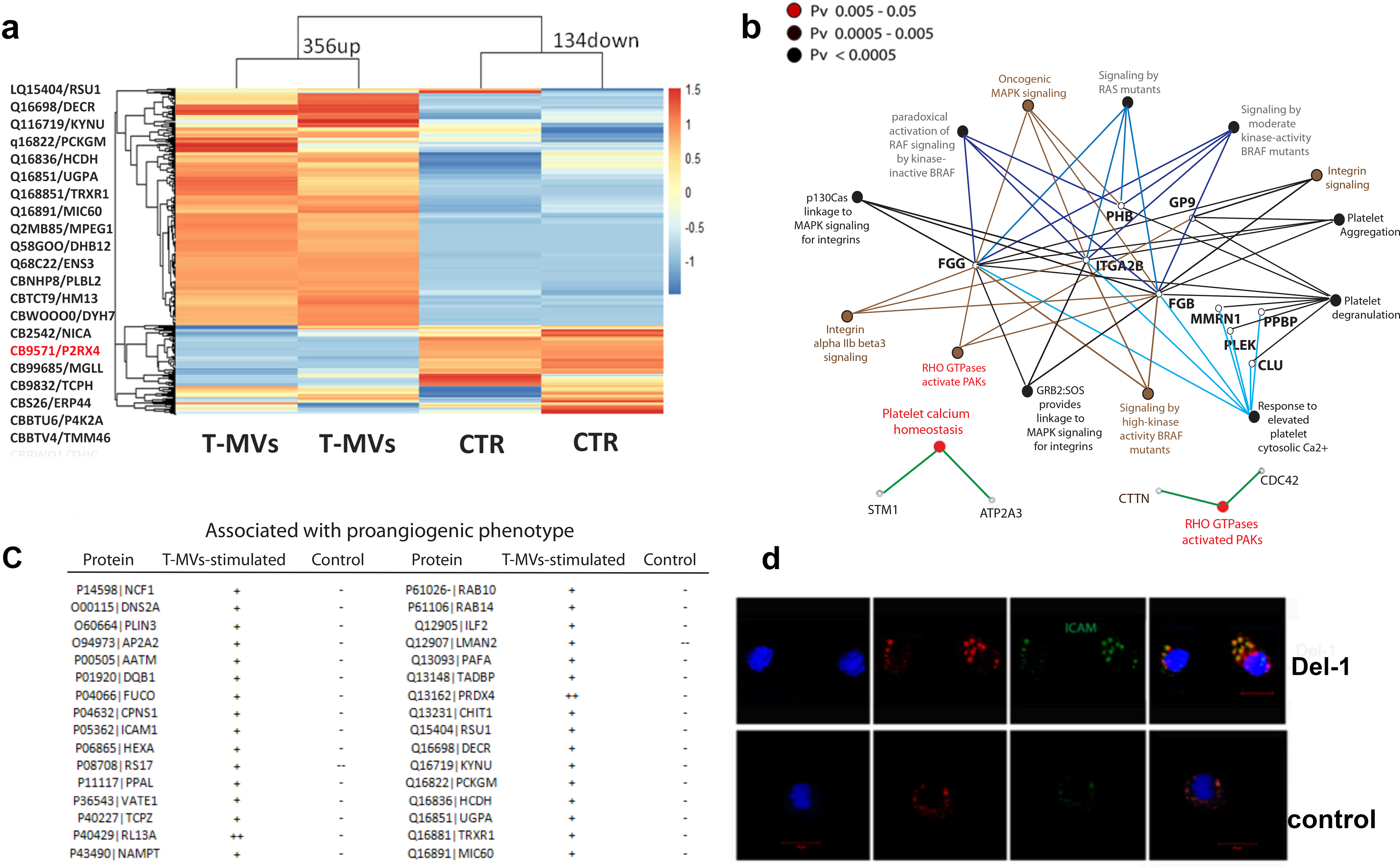

Supplement: Supplementary file 6 — Supplementary Figure 4 [file 41419_2021_4069_MOESM6_ESM.tif]

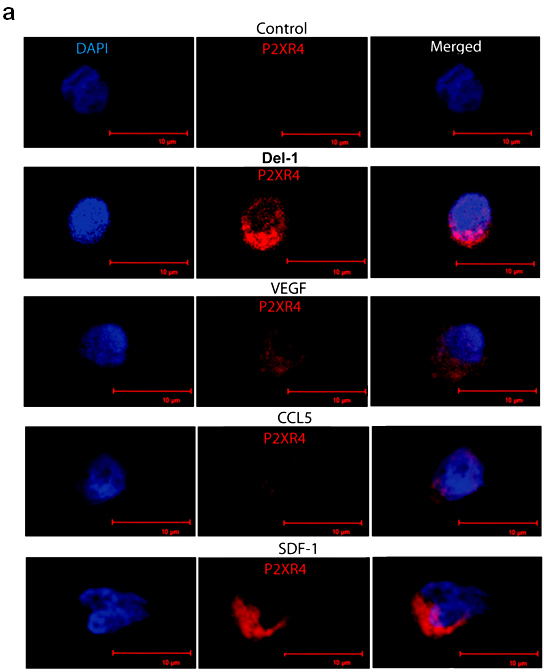

Supplement: Supplementary file 7 — Supplementary Figure 5 [file 41419_2021_4069_MOESM7_ESM.tif]

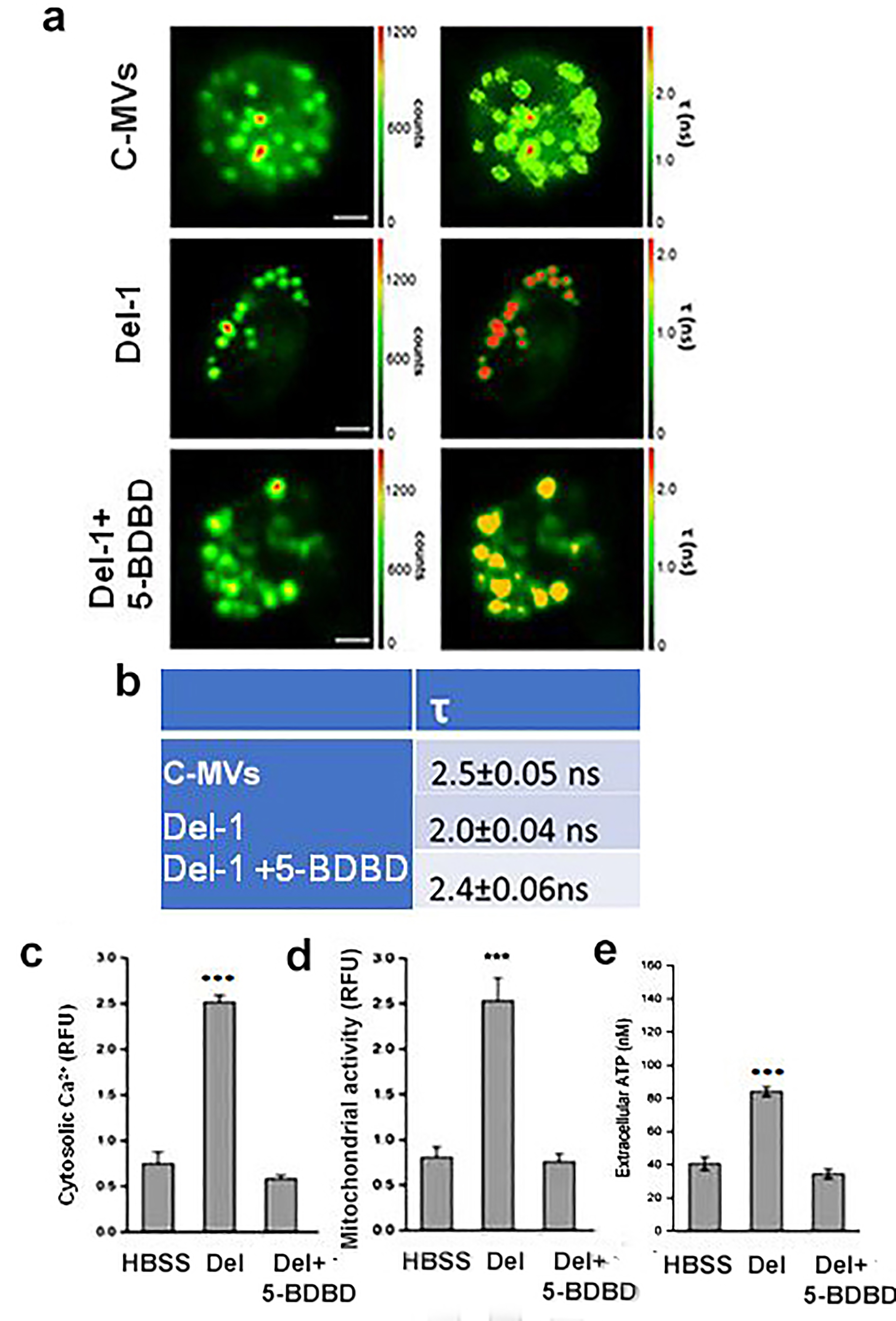

Supplement: Supplementary file 9 — Supplementary FIgure 7 [file 41419_2021_4069_MOESM9_ESM.tif]

HSP70

70KDa

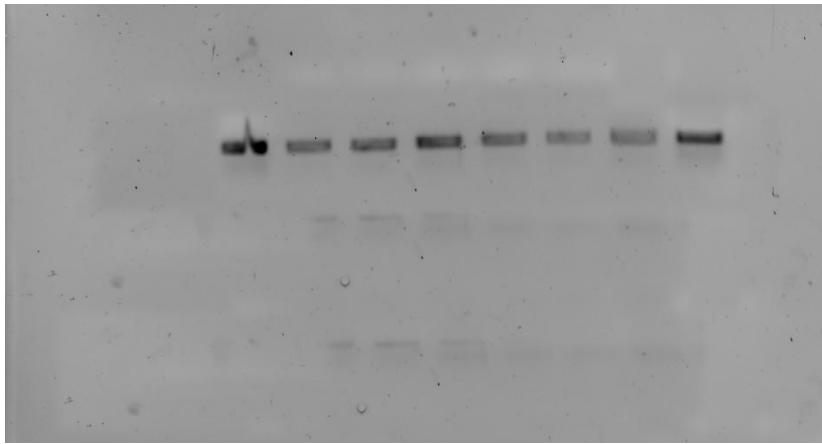

Tubulin

50KDa

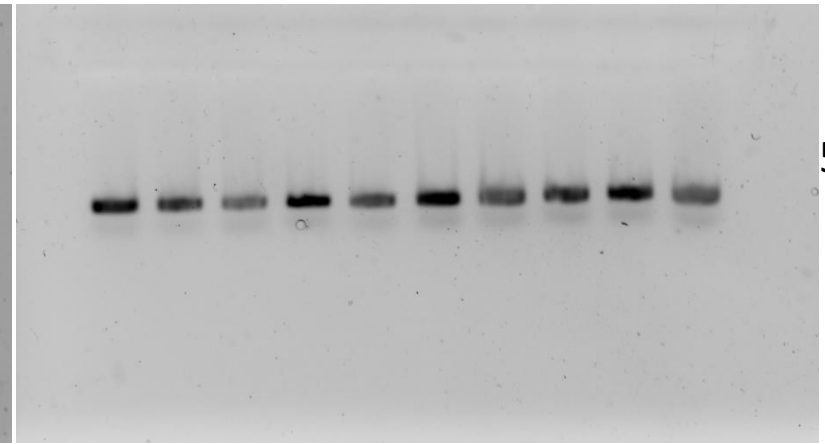

CD9

25KDa

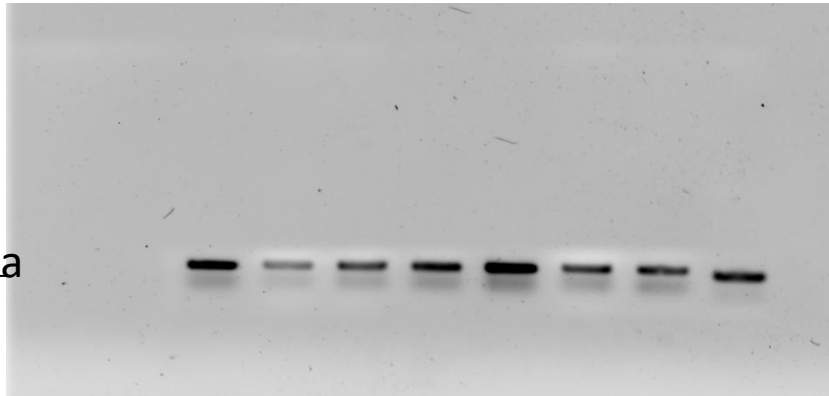

CD63

43KDa

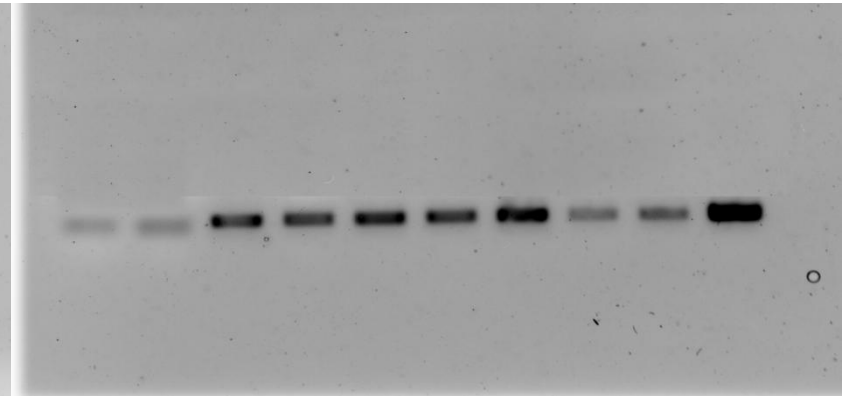

55KDa

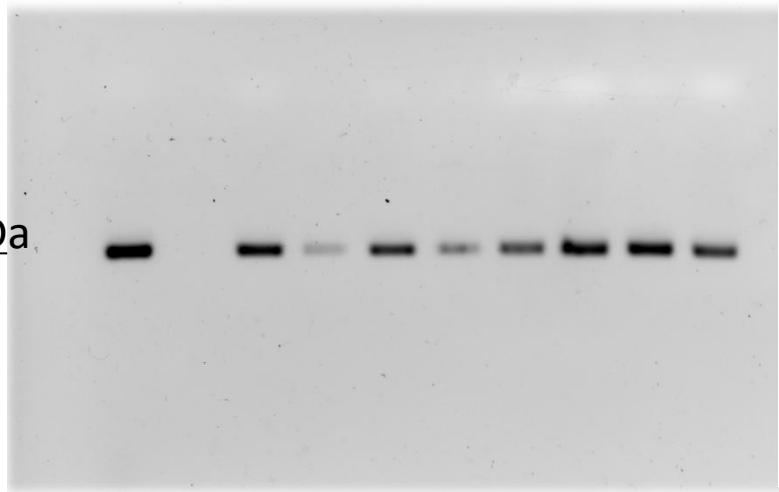

Edil3

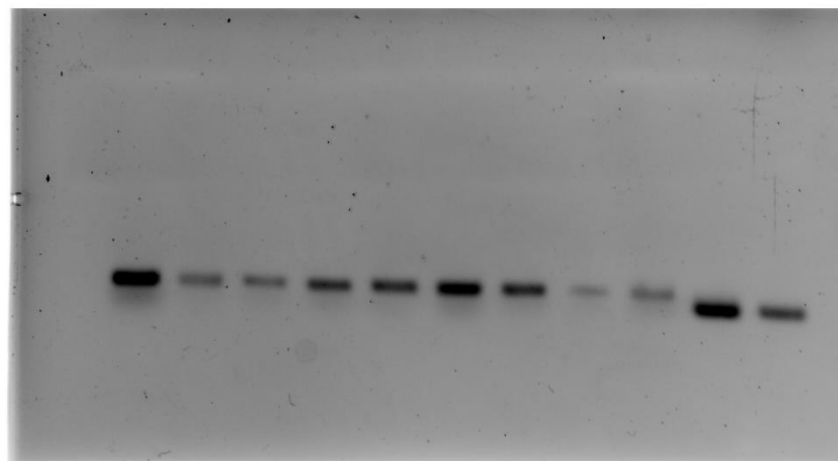

50KDa

Tubulin

Supplement: Supplementary file 14 — Data set 1 [file 41419_2021_4069_MOESM14_ESM.pdf]
