# Supplementary material for: Lysosome purinergic receptor P2X4 regulates neoangiogenesis induced by microvesicles from sarcoma patients
Source: Cell Death Dis. 2021 Aug 17;12(9):797. doi: 10.1038/s41419-021-04069-w (PMC8371002; doi:10.1038/s41419-021-04069-w)
Supplement: Supplementary file 11 — Supplementary Table 1 [file 41419_2021_4069_MOESM11_ESM.docx]

# Supplementary Table 1. Patients and Microvesicles

| **Sample** | **Patient (number)** | **MVs size (radius) (nm; mean ± SD)** | **Particles secreted (109/ml)** | **Angiogenic effect** |
| --- | --- | --- | --- | --- |
| Plasma MVs from Healthy controls | 3 | 83 ± 12 | 2.5 | no |
| Plasma MVs from Giant cell tumors | 6 | 206 ± 42 | 114.5 ± 0.5 | 3 of 6  1 of1 |

MVs from cultured media of biopsy 1 186±23 103.5 ± 0.7 1

| GCTB Case series | n=45 |  |
| --- | --- | --- |
| Age | 17-77( mean 34) |  |
| Gender | 23 M | 22 F |
| Site | Upper extremity 12 | Lower extremity 33 |
| Dimension lesion | <5cm (n=31) | >5cm (n=14) |
| Surgical treatment | Curettage (n=15) | Resection (n=30) |
| Recurrences | 11 |  |
| Ki67 | <1% (n=15) | >1% (n=30) |
